# A Comprehensive Physiotherapeutic Rehabilitation Protocol for Malunited Post-operative Patellar Fractures: A Case Report

**DOI:** 10.7759/cureus.51252

**Published:** 2023-12-28

**Authors:** Tanvi S Mirapurkar, Manali A Boob, Shruti S Bhoge, Pratik Phansopkar

**Affiliations:** 1 Musculoskeletal Physiotherapy, Ravi Nair Physiotherapy College, Datta Meghe Institute of Higher Education and Research, Wardha, IND

**Keywords:** physiotherapy, case report, rehabilitation, anatomical reduction, tension band wiring, therapeutic rehabilitation, range of motion, proprioceptive training, muscle energy technique, patellar fractures

## Abstract

A patellar fracture is a fracture of the kneecap. The patella is a shield that plays a vital function in the biomechanics of the knee joint. Traumatic knee injuries produced by direct trauma or quick quadriceps contraction with the knee bent can result in the loss of the extensor mechanism. A 69-year-old female patient presented with pain in her right knee following a slip at her home, which resulted in an injury to her knee. Subsequently, a radiological investigation suggests a patellar fracture. The purpose of this case report is to investigate the rehabilitation techniques and concrete the result. This report emphasizes the value of a comprehensive rehabilitation program designed to assist people with this type of fracture pattern in reaching their peak operational capacity. The patient underwent a comprehensive rehabilitation regimen that included cryotherapy, range of motion exercises, strengthening exercises, gait training, balance and proprioception training. The functional outcomes were assessed using a visual analogue scale, goniometry, manual muscle testing, Berg balance scale and lower extremity functional scale. The patient exhibited significant improvement and a positive response to the therapeutic techniques and outcome assessments. This underscores the necessity for a well-rounded treatment approach to manage patellar fractures and optimize patient outcomes effectively.

## Introduction

The largest sesamoid bone in the human body is the patella. The patella performs three vital roles in the knee: it guards the front of the knee, acts as a pulley to maximize the effectiveness of the extensor mechanism, and serves as an attachment point for the quadriceps tendons [1]. There is a thick layer of cartilage covering the patella [2]. These fractures typically arise from either a direct impact on the knee or the application of excessive tension on the extensor mechanism. Patella fractures can present as simple, involving bone fragments that haven't shifted from their original positions, or complex fractures where bone fragments are displaced, often necessitating surgical intervention [3]. The primary focus in patellar fracture is on two key objectives, restoring the integrity of the patellar articular surface and repairing the disrupted knee extensor mechanism. In some instances, uncomplicated patella fractures can be managed conservatively with a cast or splint until the bone heals. During the recovery period, patients are advised to refrain from activities that place undue strain on the knee joint, such as climbing stairs, squatting, or kneeling [4]. Multiple methods are available for the fixation of patellar fractures, including screw fixation. In this scenario, a combination of cerclage wire and an anterior tension band wire looping through the quadriceps tendon was employed to enhance fixation strength [5]. Physiotherapy plays a crucial role in making patients ready for activities of daily living [6]. The case report shows the plan of rehabilitation strategies for the female who was operated on for the patellar fracture. By assessing and tracking the progress of patients undergoing physiotherapy treatment, it provides valuable insights into the rehabilitation process and its impact on post-fracture outcomes.

## Case presentation

Patient information

A 69-year-old woman came to the Orthopaedic Department reporting her primary concern as right knee pain and swelling persisted for the past 15 days. The patient reported a slip at her home, which was followed by an abrupt onset of pain and progressive swelling. The pain was narrated as a dull ache and localized to the right side of the patella. There was no instance of injury to the head or bleeding from the ear, nose, or throat. There is no known relevant past medical, psycho-social, or family history to consider. Informed consent was acquired before undergoing the physical examination while lying in the supine position. On inspection, there were no scars or sinuses present; only diffuse swelling was present all over the joint, and no dilated veins were seen. On palpation, inspectory findings were confirmed. The temperature of the limb was normal. Tenderness was detected in the area above the patella. The patient was not able to flex the knee, and a crepitus sound was present. He had difficulty performing straight leg raises. There were no positive neurological tests.

Radiological findings

**Figure 1:**
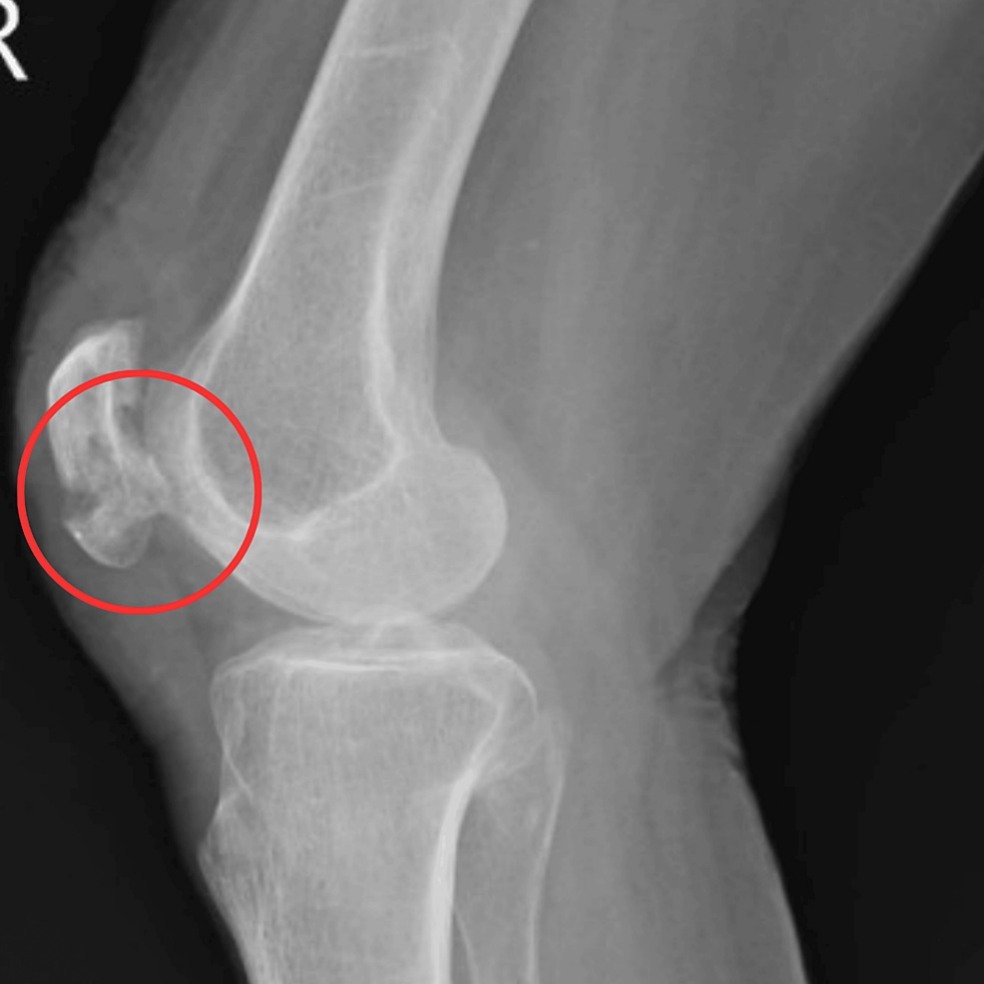
X-ray of the pre-operative patella The red circle shows the fracture of the patella.

**Figure 2:**
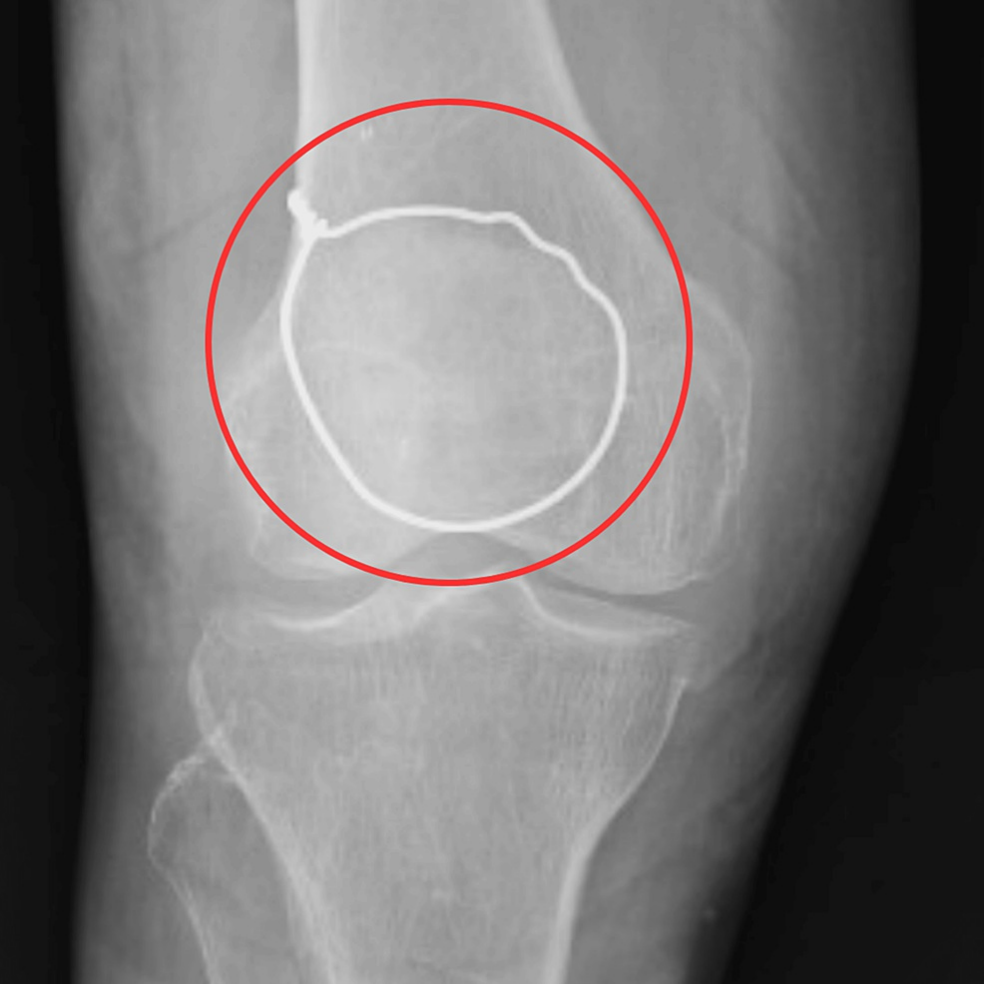
X-ray of the post-operative patella The red circle shows the fixation of fractured patellar fragments with Kirschner wire.

Radiological assessment revealed a malunited fracture of the right patella, prompting surgical intervention. Figure 1 displays the X-ray image of an undisplaced transverse patellar fracture, and Figure 2 shows the insertion of the encircle wires that were tightened.

Clinical examination

Tables 1, 2 show manual muscle testing and range of motion findings pre-rehabilitation, respectively. The power of muscles of the right side lower limb was found to be decreased. The range of motion of joints of the right lower limb was decreased as well. The assessment was done on post-operative day four.

**Table 1:** Pre-rehabilitation MMT findings MMT: manual muscle testing MMT Grades - 1: flicker of contraction, 2: full range in gravity eliminated plane, 3: full range of motion against gravity, 4: full range of motion against gravity with minimal resistance, 5: full range of motion against gravity with maximum resistance.

Joint	Right	Left
Hip flexors	2	4
Hip extensors	1	4
Hip abductors	2	4
Hip adductors	2	4
Knee flexors	2	3
Knee extensors	2	3
Ankle dorsiflexors	2	4
Ankle plantarflexor	2	3

**Table 2:** Pre-rehabilitation findings of AROM AROM: active range of motion

Joint	Right	Left
Hip flexion	0^o^-50^o^	0^o^-120^o^
Hip extension	0^o^-10^o^	0^o^-30^o^
Hip abduction	0^o^-10^o^	0^o^-45^o^
Hip adduction	0^o^-10^o^	0^o^-30^o^
Lateral rotation	0^o^-10^o^	0^o^-45^o^
Medial rotation	0^o^-10^o^	0^o^-40^o^
Knee flexion	0^o^-50^o^	0^o^-135^o^
Knee extension	50^o^-0^o^	135^o^-0^o^
Ankle plantarflexion	0^o^-10^o^	0^o^-50^o^
Ankle dorsiflexion	0^o^-10^o^	0^o^-20^o^
Ankle inversion	0^o^-15^o^	0^o^-35^o^
Ankle eversion	0^o^-5^o^	0^o^-15^o^

Therapeutics intervention

The patient began physiotherapy after the four days of the operation. The phase-wise physiotherapy rehabilitation is mentioned in Table 3. The rehabilitation started with bed mobility and progressed to proprioceptive training.

**Table 3:** Rehabilitation following a surgical procedure

Goals	Physical therapy intervention
Instruction for patient	The patient needs to be instructed about the physiotherapy plan that will be administered. Emphasize the importance of refraining from putting weight on the injured limb to prevent additional fracture displacement, which can lead to knee stiffness and complications. Ensure the patient understands the need for achieving a sufficient range of motion to avoid common complications.
In order to reduce discomfort and inflammation	Apply a crepe bandage starting from the far end and moving toward the body, follow it with cryotherapy from the far end to the near end for a duration of fifteen to twenty minutes. Conclude with a five-minute deep friction massage, utilizing a rotational friction technique to reduce swelling.
In order to enhance the extent of motion	Initiate the rehabilitation process with mild exercises aimed at maintaining joint mobility and preventing stiffness. Progressively work towards improving knee flexion, gradually progress to improve knee flexion, first targeting the range of 30 to 60 degrees, and then aiming for 90 degrees. During these exercises, active assistance in knee flexion and extension is permitted, always ensuring the knee's protection against stresses in the valgus and varus directions. Employ the Muscle Energy Technique for the quadriceps and hamstring muscles, adjusting as per the patient's tolerance while adhering to the principles of Reciprocal Inhibition. Incorporate patellar mobilization exercises into the program to facilitate the restoration of patellar mobility. Furthermore, introduce passive graded mobilization techniques.
In order to enhance muscle strength	Initiate with mild isotonic exercises for the ankles. Perform isometric Quadriceps exercises. Incorporate isometric Hamstring exercises. Isometric exercises from multiple angles to regain Quadriceps muscle mass and strength. Focus on strengthening the Vastus medialis oblique. Incorporate a range of gluteal exercises to support the patient in the transition from a seated to a standing position. While maintaining hip extensor strength. Include the clamshell exercise to enhance abduction and lateral rotation. Transition to more challenging open chain exercises that include lifting the leg while lying down, extending the knee while sitting, abducting the hip in a supine position, adducting the hip in a supine position, extending the hip in a prone position, and performing Hamstring curls while in a prone position.
Gait training	Maintain non-weight-bearing walking for the initial four weeks. Progress to touch-down weight bearing between the fifth and sixth weeks. Introduce partial weight bearing after the sixth week, gradually increasing the load on the affected limb. Initiate full weight bearing after a 12-week post-operative period. Focus on Gait Retraining phase is dedicated to refining the patient's walking mechanics. It encourages the patient to progressively increase both distance and speed, all while the general quality of one's walking. Transition to Advanced Training and Functional Activities phase comes into play after the initial 14 weeks, introducing more demanding exercises like walking on diverse surfaces, conquering stair climbing, and efficiently navigating through various obstacles.
Enhancing proprioception through training	These components are integrated into the post-fracture rehabilitation program has reached a stage of healing where the patient can comfortably bear weight. Balance exercises progress from foundational tasks such as maintaining balance on one leg with eyes open to more intricate challenges. These may encompass activities like balancing on one leg with eyes closed, engaging in activities like tossing and catching a ball while maintaining equilibrium on the impacted leg. Utilize Proprioceptive Neuromuscular Facilitation techniques tailored for the lower limb to enhance strength. Coordination drills integrate exercises such as lateral backward-forward lunges to enhance overall coordination. Exercises for Joint Position Sense engaging in active movement of the impacted joint. Throughout its complete range of motion, with the objective of replicating specific positions without relying on visual cues. These exercises play a pivotal role in honing proprioceptive skills, making the body to precisely perceive the joint's position.

Phase 1: In-Patient Rehabilitation (0-1 Weeks)

Educate the patient about physiotherapy rehabilitation. To prevent bed sores use of an air mattress, to maintain strength and mobility, exercises to improve joint strength training and range of motion exercises were started. Explain about the physiotherapy, what to do’s and don’ts perform in this condition. To prevent another complication, or secondary complications. Careful attention was given to ensuring that the ankles were positioned correctly, and that early mobility was encouraged to prevent the risk of pressure sores. Furthermore, to help maintain the strength of the lower limb muscles, a regimen of isometric exercises for the quadriceps, hamstring, and glutes was recommended [7].

Phase 2: Out-Patient Rehabilitation (2-4 Weeks)

**Figure 3:**
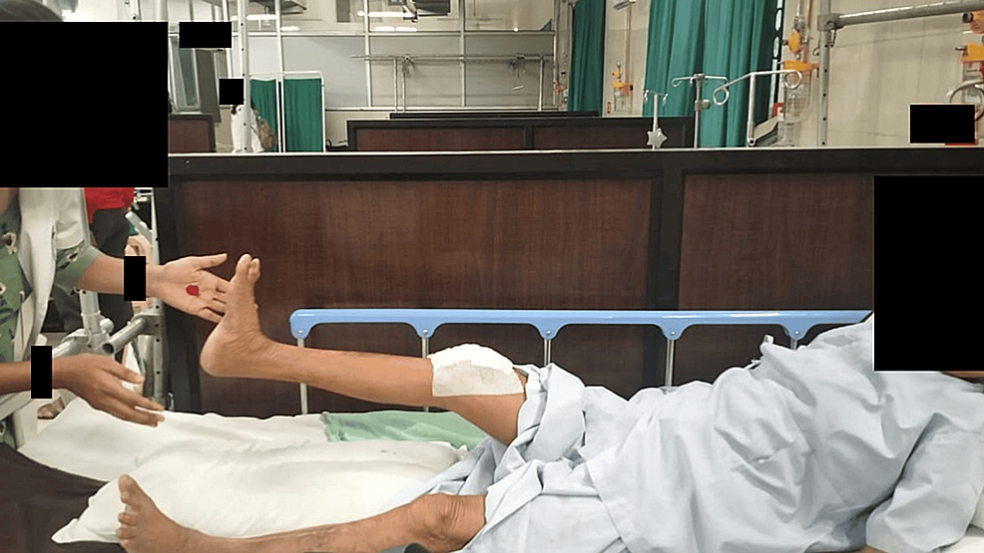
The patient performing a straight leg raise

During the second phase, which spanned 2-4 weeks, the objectives were to sustain the progress made in the initial phase while simultaneously enhancing the range of motion. To achieve this, the treatment regimen commenced with an active-assisted range of motion exercises for the knee and ankle. Subsequently, it was advanced to active range of motion exercises (Figure 3). This phase also marked the introduction of bedside sitting, accompanied by the performance of dynamic quadriceps exercises. Additionally, strength-building exercises were incorporated into the patient's routine, including pelvic bridging training and isometric exercises targeting the back and abdominal muscles [8].

Phase 3: (5-8 Weeks)

In this stage, the primary objective was to reestablish the patient's ability to take daily activities and improve their overall health. The exercises from the first and second phases were maintained and continued. In the third phase, the focus was on achieving ambulation, with advancing from partial weight-bearing to eventually achieving full weight-bearing, progress to the balancing and proprioceptive training [9,10].

Outcome measures

Treatment outcomes were recorded before treatment and were again noted during follow-up i.e., after eight weeks. Outcome measures were the visual analogue scale, range of motion, Scar scale of Vancouver, Berg balance scale and lower extremity function scale. All the measures showed considerable improvement after eight weeks of treatment as shown in Table 4.

**Table 4:** Pre- and post-rehabilitation findings of outcome measures

Measures of parameters	Pre-rehabilitation	Post-rehabilitation
Resting state Visual Analogue Scale rating	6/10	2/10
Rating of pain on a Visual Analogue Scale during knee movement	9/10	3/10
Scare scale of Vancouver	9/13	4/13
Range of knee flexion	0^O^-30^O^	0^O^-100^O^
Berg Balance Scale	13/56	46/56
Lower extremity function scale	26.2%	63.3%

## Discussion

In this case report, the management of a patellar fracture in a 69-year-old female patient using tension band wiring. The central goal of physiotherapy was restoring the patient's independence and minimizing any potential follow-up problems. To address this, a comprehensive rehabilitation protocol was established, encompassing isometric exercises, dynamic quadriceps exercises, bed mobility exercises, and ambulation training [11].

Patellar fractures, particularly malunion fractures, represent the most prevalent type of fracture in this context. The most common complications associated with such fractures are non-unions. The treatment described here was administered during the initial two weeks post-fracture to prevent complications, reestablish complete knee flexion, control oedema, facilitate effective gait education, and enhance quadriceps muscle strength [12]. Throughout the healing process, muscle strength was consistently maintained through a combination of muscular contractions that are isometric, isotonic, and isokinetic. The application of muscle energy techniques contributed to increased flexibility and strength, concurrently reducing discomfort and broadening the range of motion in the lower leg [13]. Physiotherapy plays a pivotal role in preserving and improving mobility and strength, particularly during the challenging post-operative phase [14]. The treatment sessions were geared towards maintaining muscle integrity and enhancing lower extremity exercises, such as using a walker to assist with non-weight-bearing walking and requiring little assistance with daily tasks [15]. This kind of manual therapy, called the muscle energy technique, helps to strengthen and extend the range of the movements. Physiotherapy can assist in challenging post-operative situations by maintaining and enhancing strength and mobility [16]. The objectives of the therapy sessions included minimum assistance with everyday tasks, non-weight-bearing walking with a walker, and maintaining muscle integrity while increasing lower extremity skills [17].

## Conclusions

Patellar fractures are relatively common, but their management is crucial, particularly considering the patella's mobile nature. A well-structured rehabilitation protocol is essential for successful recovery. The rehabilitation program was divided into several phases, including elements like initial immobilization, pain control, range-of-motion workouts, strength training, balance training, and assessing functional conditions. The patient moved through these phases in accordance with their degree of comfort, their ability to perform, and the radiographic findings. The patient showed outstanding throughout the rehabilitation process and functional abilities protocol. They were completely capable of handling everyday duties, including those involving the lower leg, on their own.

## References

[1] Ong TK, Chee E, Wong C, Thevarajan K (2008). Fixation of comminuted patellar fracture with combined cerclage and tension band wiring technique. Malays Orthop J.

[2] Kakazu R, Archdeacon MT (2016). Surgical management of patellar fractures. Orthop Clin North Am.

[3] Melvin SJ, Mehta S (2011). Patellar fractures in adults. J Am Acad Orthop Surg.

[4] Sun Y, Sheng K, Li Q, Wang D, Zhou D (2019). Management of comminuted patellar fracture fixation using modified cerclage wiring. J Orthop Surg Res.

[5] Schulte LM, Meals CG, Neviaser RJ (2014). Management of adult diaphyseal both-bone forearm fractures. J Am Acad Orthop Surg.

[6] Kamal A, Dong RJ, Shah R, Li C (2020). Management of periprosthetic fractures of knee arthroplasty with revision surgery. J Orthop.

[7] Kamble S, Yadav T (2020). Effect of dynamo-static splint on post operative knee stiffness. Indian J Physiother Occup Ther.

[8] Pritchett JW (1997). Nonoperative treatment of widely displaced patella fractures. Am J Knee Surg.

[9] Della Rocca GJ (2013). Displaced patella fractures. J Knee Surg.

[10] Fu FH, Rabuck SJ, West RV, Tashman S, Irrgang JJ (2019). Patellar fractures after the harvest of a quadriceps tendon autograft with a bone block: a case series. Orthop J Sports Med.

[11] Buezo O, Cuscó X, Seijas R, Sallent A, Ares O, Álvarez-Díaz P, Cugat R (2015). Patellar fractures: an innovative surgical technique with transosseous suture to avoid implant removal. Surg Innov.

[12] Ferrer MA, Lobo MO, Almeida LM, Freitas A, Macedo Neto SL, Paiva LM, Battaglion LR (2023). Patellar fracture in anterior cruciate ligament reconstruction: in vitro analysis. Acta Ortop Bras.

[13] Harna B, Gupta P, Singh J, Rousa S, Gupta A (2021). Surgical management of non-union patella fracture: a case series and review of the literature. Arch Bone Jt Surg.

[14] Govil G, Tomar L, Dhawan P (2020). Peri-prosthetic trans-patellar fractures after total knee arthroplasty: a case series and review of literature. Arthroplasty.

[15] Stein DA, Hunt SA, Rosen JE, Sherman OH (2002). The incidence and outcome of patella fractures after anterior cruciate ligament reconstruction. Arthroscopy.

[16] Levack B, Flannagan JP, Hobbs S (1985). Results of surgical treatment of patellar fractures. J Bone Joint Surg Br.

[17] Braun W, Wiedemann M, Rüter A, Kundel K, Kolbinger S (1993). Indications and results of nonoperative treatment of patellar fractures. Clin Orthop Relat Res.

